# “Doc McStuffins: Doctor for a Day” Virtual Reality (DocVR) for Pediatric Preoperative Anxiety and Satisfaction: Pediatric Medical Technology Feasibility Study

**DOI:** 10.2196/25504

**Published:** 2021-04-19

**Authors:** Jeffrey I Gold, Erin T Annick, Arianna S Lane, Katherine Ho, Ryan T Marty, Juan C Espinoza

**Affiliations:** 1 Department of Anesthesiology Critical Care Medicine The Saban Research Institute Children’s Hospital Los Angeles Los Angeles, CA United States; 2 Departments of Anesthesiology, Pediatrics, and Psychiatry & Behavioral Sciences Keck School of Medicine University of Southern California Los Angeles, CA United States; 3 Division of General Pediatrics, Department of Pediatrics The Saban Research Institute at Children’s Hospital Los Angeles The West Coast Consortium for Technology & Innovation Pediatrics Los Angeles, CA United States; 4 Department of Pediatrics Keck School of Medicine University of Southern California Los Angeles, CA United States

**Keywords:** virtual reality (VR), pediatric, anxiety, preoperative, satisfaction, Doc McStuffins

## Abstract

**Background:**

Preoperative anxiety is a common occurrence among children and is associated with a host of maladaptive postoperative behaviors. Consequently, increased attention has been placed on interventions to reduce preoperative anxiety and its associated outcomes. Child Life preparation prior to surgery includes evidence-based practices such as age-appropriate distraction and therapeutic play. Virtual reality (VR) is a promising addition to the Child Life toolbox to address anxiety prior to surgery. The current study evaluates the implementation and feasibility of a VR experience, “Doc McStuffins: Doctor for a Day Virtual Reality Experience” (*Doc*VR), developed by Disney Junior in collaboration with Children’s Hospital Los Angeles, to target pediatric preoperative anxiety.

**Objective:**

The primary aim of this study was to examine the feasibility and efficacy of *Doc*VR for preoperative anxiety. A secondary aim was to improve patient, caregiver, and health care provider satisfaction with the preoperative experience.

**Methods:**

In this study, 51 patients (age 6-14 years) scheduled for surgery in the ambulatory surgery center and the main operating room at Children’s Hospital Los Angeles were approached to participate in Disney’s *Doc*VR experience. The patients played the *Doc*VR experience for an average of 18 minutes (3-55 minutes). Irrespective of surgical procedure, patients and their families were eligible, as long as they had no known marked cognitive or visual impairments that would interfere with completing the survey and engaging in the *Doc*VR experience.

**Results:**

Patients who tried the *Doc*VR experience (n=51) responded overwhelmingly positively to both the VR technology and to the game itself. Patients experienced a statistically significant decrease in anxiety following DocVR game play (Z=–3.26, *P=*.001). On the Facial Affective Scale, the percentage of patients who chose the face with the most positive facial expression to represent their affect increased from 23% (12/51) pre-VR to 49% (25/47) post-VR. Furthermore, 97% (38/39) of patients reported feeling more comfortable at the hospital, and 74% (28/38) reported feeling less scared at the hospital after playing the game. The game was enjoyed by 94% (46/49) of patients, and 88% (30/34) of patients reported feeling both “Interested” and “Involved” in the game.

**Conclusions:**

*Doc*VR is a feasible and beneficial VR experience to relieve pediatric preoperative anxiety and improve satisfaction in the preoperative area. The VR experience resulted in a decrease in overall anxiety and an increase in overall positive affect during the preoperative time. Patients also responded positively to the game, confirming their interest in the content and affirming the quality of the *Doc*VR experience. The positive response to the game indicates that *Doc*VR has the potential to make the overall preoperative experience less anxiety-producing and more comfortable, which leads to improved patient satisfaction. Naturally, improved patient outcomes lead to improved caregiver and health care provider satisfaction.

## Introduction

Nearly 5 million children undergo surgery in the United States annually, and 50-75% of these children experience preoperative anxiety [1]. Preoperative anxiety not only causes distress and suffering in children prior to surgery but is also associated with a slower, more painful recovery and negative postoperative behavior changes, such as separation anxiety, sleep disturbances, eating difficulties, and aggression towards authority [2,3]. Additionally, children who experience high preoperative anxiety are more likely to develop emergence delirium, a state of dissociated consciousness characterized by uncooperativeness, inconsolable crying, irritation, and incoherency that occurs upon waking from anesthesia [4,5].

Techniques for reducing preoperative anxiety in pediatric patients fall into 4 broad categories: preoperative sedatives, parental presence, preparation programs [6], and pain or anxiety management interventions. Although preoperative sedatives are regularly administered prior to surgery, anti-anxiety drugs can synergistically interact with anesthetics to produce undesirable side effects that prolong recovery [7]. Additionally, the use of sedatives may delay hospital discharge and consequently increase operational costs [6,8,9]. To date, research on the effect of parental presence for preoperative anxiety has yielded mixed results; although some studies have documented its efficacy, other studies have found no significant differences between anxiety levels of children whose parents were present versus absent at various stages of the preoperative process [10]. Thus, clinicians and researchers have long been interested in using other nonpharmacological interventions, such as procedural preparation programs and anxiety and stress management interventions, to combat preoperative anxiety. These combination interventions, which include Child Life programs and virtual reality (VR), among others, can work in tandem to provide potentially greater reductions in pediatric preoperative anxiety and distress, thus ultimately diminishing known features associated with medical trauma.

A variety of health care providers, including nurses, physicians, and psychologists, deploy VR for an array of health care–related interventions. In the preoperative space, Certified Child Life Specialists (CCLSs) are often the frontline professionals providing preparation and anxiety management strategies for patients in pediatric facilities. CCLSs are professional health care providers who help patients and their families cope with the challenges of the medical world, including, but not limited to, initial diagnoses, illness, hospitalization, and medical procedures [11]. CCLSs have spent decades continuously adding new innovations to their toolboxes to help children and families manage pain and anxiety associated with medical procedures [12-14]. For example, CCLSs capitalize upon the gate control theory of pain to help children cope with the unpleasant sensations caused by medical environments [15]. By providing engaging distractions like age-appropriate toys, electronic devices, and deep breathing techniques, CCLSs can divert the child’s attention away from their upcoming procedure and close the child’s “gate” for pain sensation. CCLSs also recognize the role of mental preparation and education in reducing preprocedural pain and anxiety. Numerous studies have shown that preparation programs can significantly reduce children’s negative responses to medical procedures [16-20]. Mentally preparing patients and their families for upcoming procedures can involve giving tours of the operating room (OR), allowing children to familiarize themselves with medical equipment, and facilitating medical play sessions with dolls [16]. Whether providing distraction or education, CCLSs help align the perspectives of patients, caregivers, and health care providers to mitigate the oftentimes debilitating stresses of the medical environment.

VR has emerged as a promising nonpharmacological intervention to relieve pediatric preoperative anxiety and improve satisfaction. Incorporation of visual, auditory, and tactile stimulation within a 3D environment allows a child to “escape” to another world. The current study utilized a head-mounted display VR technology, which is the most common mode of technology for VR intervention-based studies. VR has been shown to reduce both pain and anxiety in pediatric populations for a variety of procedures, including phlebotomy [21], peripheral intravenous catheterization access [22], invasive dental procedures [23], and wound care and dressing changes [24]. A majority of pediatric VR studies for anxiety management focus on periprocedural timeframes, as cited earlier in this manuscript, and only a very limited number of studies have examined the use of VR for preoperative anxiety [25,26]. Additionally, there has been a call for a greater focus on anxiety management in VR research [27]. The current study’s focus on both the preoperative timeframe and anxiety management makes it particularly relevant as VR research continues to charge forward.

Disney Junior, a television network owned by The Walt Disney Company, is a familiar staple in children’s entertainment. Their television shows and online interactive games keep children engaged while reiterating life lessons of bravery, kindness, and friendship [28-30]. One of these shows is “Doc McStuffins: Toy Hospital,” which follows a young girl who acts out her dream of becoming a pediatrician on her toys. Cast members from multiple Disney teams created “Doc McStuffins: Doctor for a Day” (*Doc*VR), a VR experience to engage, educate, entertain, and immerse patients into a “Doctor for the Day” role, virtually conducting routine medical procedures, while waiting for their planned outpatient surgery. A multidisciplinary team from Children’s Hospital Los Angeles (CHLA), including psychology, Child Life, and general pediatrics, implemented the Disney Junior *Doc*VR experience. Considering the medical context of the Doc McStuffins show, *Doc*VR is well-matched for pediatric medical centers, as it exposes and normalizes medical experiences, all in the tried-and-true format of an already beloved show. Incorporating VR, and particularly *Doc*VR, into the CCLS’s preoperative processes has promising applications to more effectively manage preoperative anxiety.

This undertaking marks the first collaboration between Disney Junior and a children’s hospital to build and pilot, respectively, a VR experience. This study assessed the feasibility of the *Doc*VR experience for preoperative anxiety and overall presurgical satisfaction. We hypothesized that *Doc*VR would reduce patient anxiety and overall distress, while improving patient, caregiver, and health care provider satisfaction in the ambulatory surgery center (ASC) or the main OR.

## Methods

This study included data from 51 patients, ranging in age from 6 to 14 years old, collected from March 15, 2019 to April 12, 2019. Patients were accompanied by at least one caregiver in the room during the VR experience. Given the time-sensitive nature of the OR, patients were available to play *Doc*VR and to answer study questions for variable lengths of times. All activities for the current study were approved by the institutional review board.

### Recruitment

Pediatric patients waiting for surgical procedures in either the CHLA ASC or the CHLA main OR were approached to participate in the feasibility pilot. Though most patients were approached in the waiting rooms, some were approached in the preoperative holding areas. The research team worked with CCLSs in the ASC and OR to screen for and approach eligible patients.

All patients between the ages of 6 and 18 years with normal vision and typical cognitive development were eligible to participate, irrespective of surgery type. Cognitive and visual impairments rendered patients ineligible as these would interfere with completing the survey or playing the *DocVR* game. Due to the varied consensus on the minimum age for VR headsets, 6 years old was the minimum age for participation.

### Development of DocVR

#### Preparation

In 2016, Disney Junior and CHLA had a series of meetings to discuss creating a VR experience with Doc McStuffins for the purposes of entertainment and to comfort children in the hospital setting. After multiple brainstorming meetings, the Disney Junior creative staff (termed “cast members”) iterated on the VR environment and organized a demonstration day at the hospital for CHLA stakeholders. Having received feedback regarding the user experience of the prototype and what children typically enjoy, the cast members finished the virtual environment, and the team planned for a launch date, including decorating the waiting rooms for the ASC and the main OR area. The Disney team decorated the waiting areas with specific Doc McStuffins decals, transforming the virtual experience into a “real” physical space.

#### Virtual Reality

**Figure 1:**
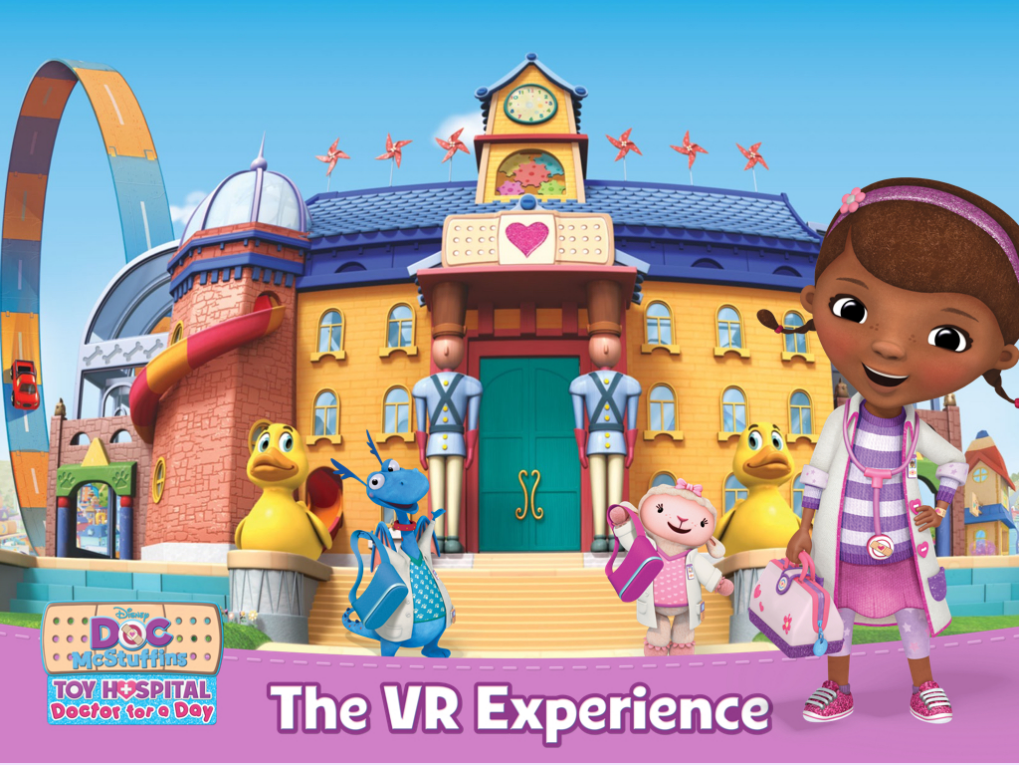
Doc McStuffins in front of the Toy Hospital along with Lambie Lamb and Stuffy.

Inside the VR experience, users choose to enter either a toy hospital (main experience; Figure 1) or a theater (supplementary experience). If the toy hospital is chosen, users help Doc McStuffins treat toy patients by completing a series of game-like VR tasks. If the theater is chosen instead, users can watch a selection of Doc McStuffins episodes and clips.

Upon choosing the toy hospital experience, users are greeted by Dottie (“Doc”) McStuffins, who instructs them to click on a set of doors to enter the virtual hospital (Figure 1). After entering the hospital, users arrive at the OR front desk, where Doc explains that users will assist as her “medical student for the day.” Users are also introduced to Nurse Hallie Hippo and fellow medical student Lambie Lamb. Users then click on a book of patients to pick 1 of 5 toy characters to treat.

**Figure 2:**
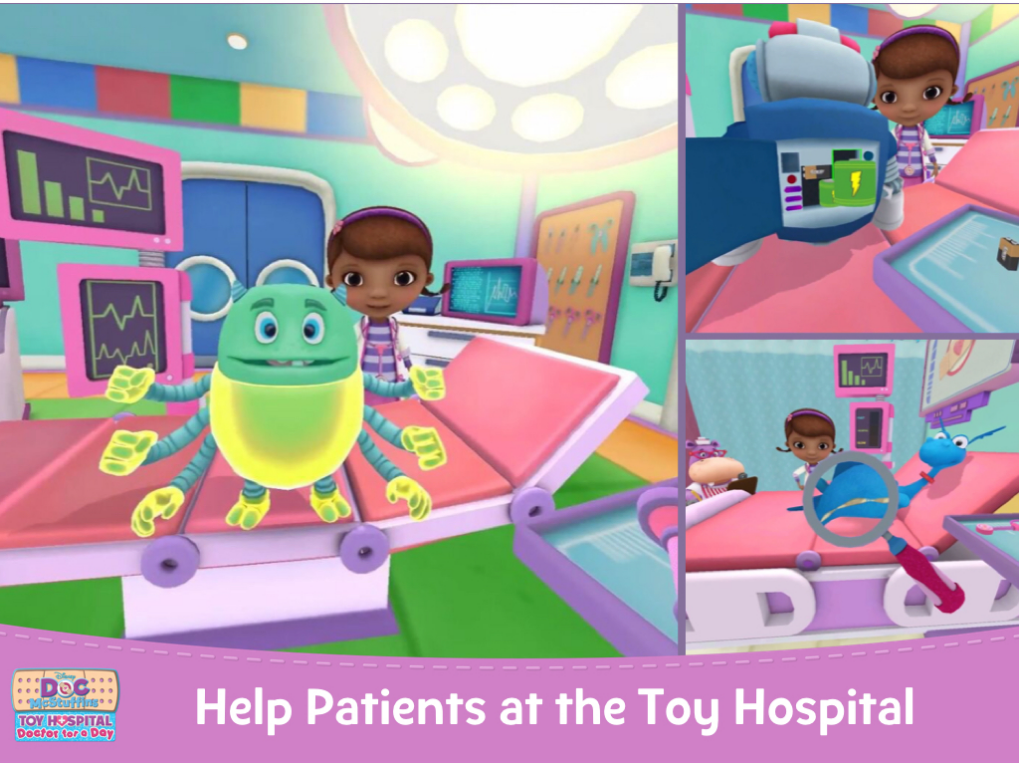
A few examples of what a user sees when playing DocVR Experience.

The 5 characters are a robot, Robot Ray; a blue dinosaur stuffed animal, Stuffy; a purple plastic shark, Mr. Chomp; a green toy monster, Globo; and a superhero action figure, Awesome Guy. Each character has a health issue that the user helps Doc treat (Figure 2). Robot Ray is sick with “Drainy Battery-itis,” treatment of which requires users to open Robot Ray’s back panel with a screwdriver, replace his batteries, and reclose the battery compartment door. Stuffy is afflicted with “Ripped Plush-Anemia,” healing of which requires the sewing up of a rip in Stuffy’s fur. Mr. Chomp suffers from “Stuck-Junk-itis,” for which users can remove junk objects from Mr. Chomp’s mouth. Globo has a diagnosis of “No-Glow-Atosis,” which is treated by pointing a Sunlight Power-Upper at his many moving hands for recharging. Awesome Guy is aching from “Crackety-Crackatosis,” recovery of which requires players to scrub his cracks clean and seal them with paste. Each character takes about 5 minutes to treat, amounting to a VR experience lasting up to 25-30 minutes.

Besides playing engaging games to treat the toys’ afflictions, users are also exposed to the medical day-to-day of check-ups and diagnoses. For example, they use blood pressure cuffs to record the toys’ energy levels, stethoscopes to listen to heartbeats, an x-ray machine to capture the toys’ internal features, and magnifying glasses to examine cuts, cracks, and rips. Furthermore, Doc puts some of the toys to sleep before treating them, paralleling the experience of general anesthesia in a real OR.

### Study Procedures

In collaboration with CCLSs, patients were identified and approached to pilot *Doc*VR. After confirming the patient’s interest, a research assistant administered a pre-*Doc*VR web-developed survey on Qualtrics using an iPad. Questions in the survey assessed the patient’s current levels of anxiety (Visual Analogue Scale [VAS] for Child Anticipatory Anxiety/Procedural Anxiety), current overall mood (Facial Affective Scale [FAS]), thoughts and feelings about the upcoming surgery, and familiarity with both the Doc McStuffins character and VR itself.

**Figure 3:**
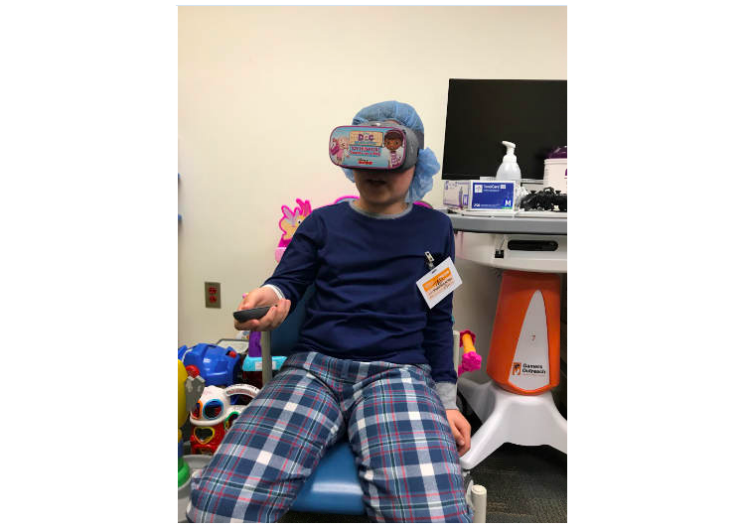
A Children's Hospital Los Angeles patient playing the DocVR Experience.

After the patient completed the pre-*Doc*VR experience survey, the research team launched the *Doc*VR application on a Google Pixel 2 phone and inserted it into the Google Daydream View VR, before fitting the headset on the patient (Figure 3). Using the handheld controllers, the patient was then instructed to click through 2 floating bandage icons to begin the *Doc*VR experience.

Following gameplay, the research assistant administered the patient’s post-*Doc*VR experience survey. The post-*Doc*VR survey, similar to the pre-*Doc*VR survey, evaluated the patient’s current/post-VR anxiety, satisfaction, and affect/emotions; issues pertaining to immersion in the *Doc*VR experience; issues related to overall satisfaction; and an opportunity for general comments and feedback about the *Doc*VR experience.

At the conclusion of the VR experience, the patient was given a Doc McStuffins–themed badge lanyard inscribed with Dr. (child’s name), sticker sheets, and toys to celebrate completing the *Doc*VR experience.

To maintain good hygiene and eliminate infection disease concerns, each patient wore a disposable felt face mask under the VR headset and a hair net. Additionally, research staff sanitized the controller and headset between each patient with Sani-Cloth Germicidal Disposable Wipes.

### Data Collection and Measures

#### VAS for Child Anticipatory Anxiety/Procedural Anxiety

The VAS anticipatory anxiety measure is a vertical VAS, anchored with 0 at the bottom indicating the least amount and 10 at the top indicating the greatest amount, in response to the instruction to rate “how nervous, afraid, or worried” they were about the upcoming medical procedure or surgery. This continuous measure also has color cues, graded from yellow at the bottom to dark red at the top, as well as a neutral face at the bottom and a face showing a negative expression at the top. Prior research has used the VAS to rate anticipatory anxiety and pain in children [19,31,32].

#### Facial Affective Scale (FAS)

The FAS is a cartoon face scale with 9 faces ranging from smiling widely (Face 1: least distressed) to crying (Face 9: most distressed) [33]. The scale measures both pain intensity and emotional affect, giving insight to the child’s overall level of discomfort, both physical and emotional. The scale can be used objectively by caregivers and health care providers, or the child can point to the face he feels represents how he feels at a given point in time.

#### Additional Questions: VR Experience Survey

A Disney Junior/CHLA-developed survey was created to assess various aspects of the patient’s experience with *Doc*VR. The questions assessed overall enjoyment of the *Doc*VR game and thoughts and feelings that occurred while playing the game. The survey also included questions that asked patients about game-specific elements, such as which characters they helped, which characters were their favorite, what they liked most and least about the game, and whether or not they found the game difficult. Patients were asked various questions about how familiar they were with both VR and Doc McStuffins before playing the game and how often they thought about their surgeries before and during the VR experience. Additionally, the post-VR survey included a 14-item questionnaire that assessed the degree of immersion in the VR experience. All self-reported answers on the immersion questionnaire were scored on a 3-point Likert Scale reflecting “A Lot,” “A Little,” or “No/Not at All.” Finally, a research assistant also noted the patient’s gender, age, and number of minutes spent playing the *Doc*VR experience.

### Statistical Analysis

Descriptive statistics were used to summarize quantitative data from patient surveys. A nonparametric Wilcoxon signed-rank test was conducted with SPSS version 26 to calculate the change in the pre- and postanxiety scores (VAS for anticipatory anxiety), since this was a continuous variable with nonnormally distributed data.

## Results

### Pre-DocVR

Though this study included 51 patients, due to the OR schedule and based on which questions patients wanted to answer, the response rates for each question varied and are noted as such.

Of the 51 patients, 76% (39/51) had heard of Disney Junior, 90% (46/51) had heard of Doc McStuffins, and 32 had watched Doc McStuffins on television. Regarding VR play, 71% (36/51) of patients had never played VR, and 29% (15/51) of patients had played VR in the past. Those patients had used VR an average of 2.4 times, but 4 patients had used it 4 or more times. Overall, the patients were highly familiar with Disney Junior and Doc McStuffins, but few had actually used VR in the past.

### Time Spent Thinking About Their Surgeries

**Figure 4:**
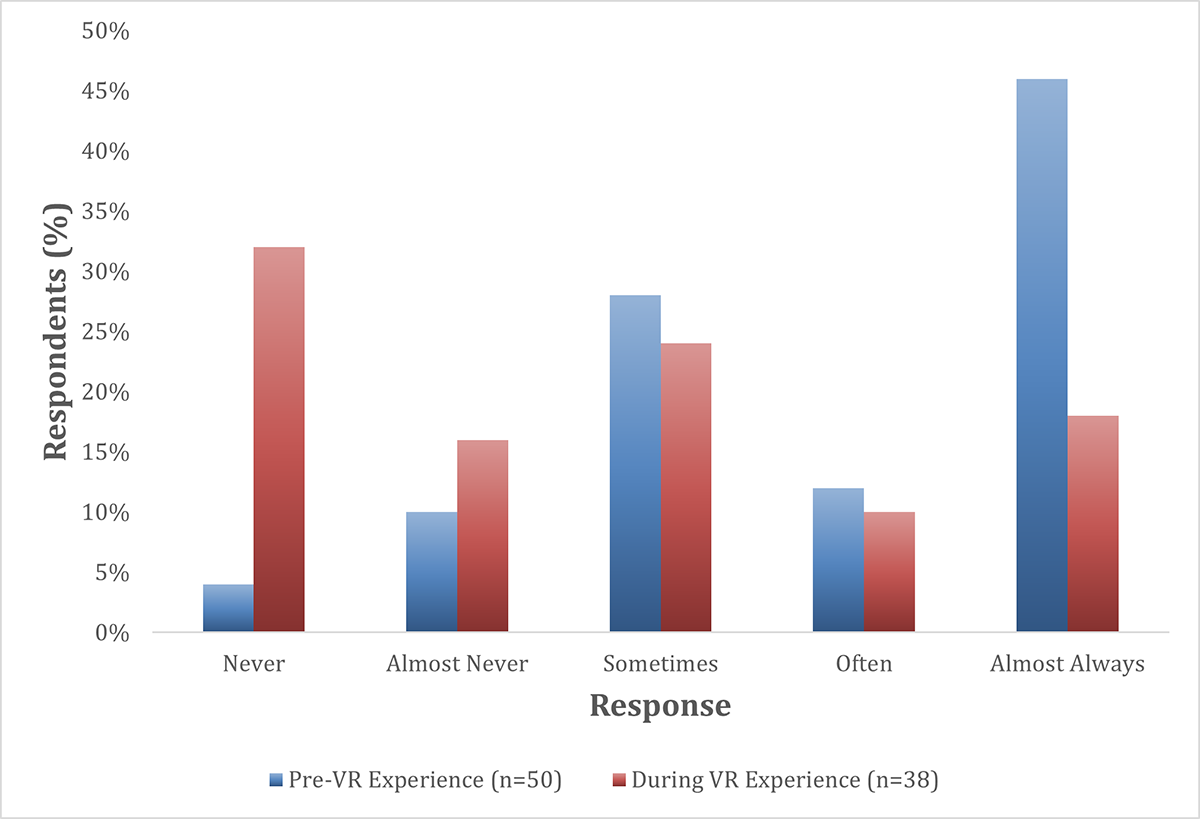
Self-reported time spent thinking about their surgery before and during the DocVR experience.

Before playing the VR experience (n=50), based on the 5-level categorical ranking of time spent that morning thinking about “today’s” surgery, 86% (43/50) of patients reported thinking about their surgery “Sometimes,” “Often,” or “Almost Always,” with just 14% (7/50) reporting “Never” or “Almost Never” (Figure 4). While playing *Doc*VR (n=38), 52% (20/38) of patients reported thinking about the surgery “Sometimes,” “Often,” or “Almost Always” while playing the game, while 48% (18/38) of patients reported that they “Never” or “Almost Never” thought about their surgery during gameplay, demonstrating a 34% decrease in “time spent thinking about [their] surgery” between the pre-*Doc*VR (n=50) and post-*Doc*VR (n=38) groups of patients (Figure 4). When patients were asked what they were thinking about during *Doc*VR, they reported a variety of statements ranging from “fun” to “nothing” (Table 1).

**Table 1:** Patients’ thoughts during gameplay with DocVR, in response to the question “What did you think about during ‘Doc McStuffins: Doctor for a Day?’”

Response	Number of patients (n=24)
“It was fun”	10
“I felt good”	4
“Playing the game”	2
“Nothing”	1
“Excited”	1
“It was cool”	1
“It was hard”	1
“Surgery”	1
“I felt like a real doctor”	1
“That Doc was helping a lot”	1
“Nervous to help patients”	1

### Pre- and Post-VR Affect and Anxiety

The median level of anxiety (n=51) reported by patients prior to *Doc*VR gameplay was 4.0 (IQR 0.60-7.43), while the median level of anxiety following gameplay (n=46) was 0.91 (IQR 0.30-4.60). Patients displayed a statistically significant decrease in anxiety after *DocVR* gameplay (Z=–3.26, *P=*.001).

**Figure 5:**
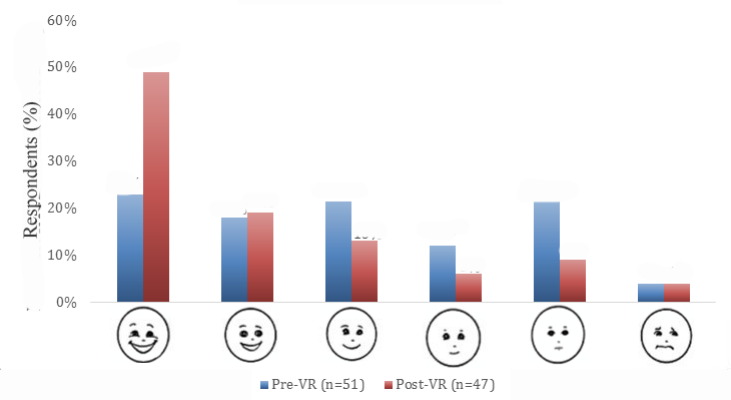
Self-reported affect pre- and post-DocVR gameplay experience. Faces 7-9 (faces that represent the 3 most distressed affects) were not selected by any participants pre- or post-DocVR and are thus not represented in the graph.

Patients reported increases in positive affect from 23% (12/51) to 49% (23/47) following *Doc*VR gameplay (Figure 5). Similarly, a breakdown of the 3 most positive faces chosen versus the 3 most negative faces chosen shifted from 63% (32/51) and 38% (19/51) to 81% (38/47) and 19% (9/47), respectively, after playing the VR experience (Figure 5). Of the 9 faces in the FAS, no participants reported Faces 7-9 (faces that represent the 3 most distressed facial affects) pre- and post-*Doc*VR.

After *Doc*VR gameplay (n=39), 97% (38/39) of patients reported that playing the game made them feel more comfortable at the hospital: 51% (20/39) reported feeling “A Lot” more comfortable, and 46% (18/39) reported feeling “A Little” more comfortable. In addition, 74% (28/38) of patients reported that playing the game made them feel less scared at the hospital, with 29% (15/38) reporting “A Lot” less scared and 45% (17/38) reporting “A Little” less scared. When patients were asked about their feelings during *Doc*VR, they reported a variety of statements ranging from “a little better” to “good” (Table 2).

**Table 2:** Patients’ feelings during gameplay with DocVR, in response to the question “How did you feel while playing ‘Doc McStuffins: Doctor for a Day?’”

Response	Number of patients (n=23)
“Good”	6
“Happy”	5
“Fun and excited”	3
“Like a real doctor”	2
“Nervous”	2
“Cool”	2
“Like I was helping”	1
“Shy”	1
“A little better, because they had to go into a little surgery too”	1

### Self-Reported Satisfaction With DocVR

On a 3-point anchor scale with responses of “No,” “A Little,” and “A Lot,” 94% (46/49) of patients enjoyed playing the game, with 76% (37/49) reporting that they enjoyed the game “A Lot,” and 18% (9/49) reporting “A Little.” Of the 29% (15/51) of all patients who had played VR before, 100% (15/15) enjoyed *Doc*VR. Additionally, 88% (30/34) of patients reported feeling both “Interested” and “Involved” in the game, while 56% (19/34) reported their levels of interest and involvement as “A Lot,” and 32% (11/34) reported it as “A Little.” Of the patients, 97% (34/35) reported that playing the game “grabbed [their] attention”; 51% (18/35) reported it did so “A Lot,” and 46% (16/35) reported it did so “A Little.”

Of the patients, 88% (28/32) reported that *Doc*VR was also interesting compared to other computer games they had played; 41% (13/32) reported the game as being “A Lot” more interesting, and 47% (15/32) reported it as being “A Little” more interesting.

Of the patients, 97% (33/34) reported that they felt like they were “really” there in the VR world; 68% (23/34) reported feeling “A Lot” like they were there, and 29% (10/34) reported feeling “A Little” like they were there.

Of the patients, 53% (18/34) reported feeling sad or disappointed when the game ended; 18% (6/34) reported the level of their feelings of sadness or disappointment as being “A Lot,” and 35% (12/34) reported the level of these feelings as “A Little.”

No children reported known side effects to VR like dizziness, nausea, or light-headedness. 

When patients were asked about they liked most about *Doc*VR, they reported a variety of statements ranging from “airplanes” to “helping people/patients/characters” (Table 3).

**Table 3:** Self-reported comments describing what the patients liked the most about the DocVR experience, in response to the question “What did you like MOST about ‘Doc McStuffins: Doctor for a Day?’”

Response	Number of patients (n=28)
“Helping patients/people/characters”	12
“Helping Mr. Chomp”	3
“Everything”	2
“Fixing Stuffy”	2
“Playing”	2
“Helping Robot Ray with the batteries”	2
“Liked the patient going to sleep so it didn’t feel any pain”	1
“It feels like I was really there”	1
“Airplanes”	1
“The surgery”	1
“The whole 3D environment”	1

### “Doctor for a Day” Implementation Feasibility

Of the patients, 88% (29/33) reported that they would like to play the game again, with 61% (20/33) reporting “A Lot” and 27% (9/33) reporting “A Little.” Of the patients, 97% (32/33) reported that they would recommend the game to another patient in the hospital or friend, with 70% (23/33) reporting “A Lot” and 27% (9/33) reporting “A Little.” Responses to other feasibility questions are shown in Table 4.

**Table 4:** Patients’ responses to a variety of virtual reality feasibility questions.

Question	Response, n (%)		
	A Lot	A Little	No
Did you get used to the game quickly? (n=38)	27 (71)	5 (13)	6 (16)
Were the controls easy to use? (n=38)	22 (58)	7 (18)	9 (24)
Did the things you saw look real? (n=33)	18 (55)	10 (30)	5 (15)
Was the headset comfortable? (n=34)	15 (44)	15 (44)	4 (12)
Were you worried about putting on the headset? (n=34)	4 (12)	3 (9)	27 (79)
Did it feel like you were in control? (n=34)	18 (53)	10 (29)	6 (18)
Did the way things moved look real? (n=33)	17 (52)	11 (33)	5 (15)

## Discussion

The primary aim of this study was to assess the usability and feasibility of *Doc*VR*,* a novel, interactive, fully immersive 3D experience developed by Disney Junior and implemented by CHLA.

### Key Findings

Findings suggest that the *Doc*VR is feasible, effective, and enjoyable and shows promise as a tool to alleviate pediatric preoperative anxiety and improve overall patient, caregiver, and health care provider experience during the preoperative period.

*Doc*VR significantly decreased patients’ anxiety, leading them to think about things other than their apprehension about their procedures. Additionally, the virtual hospital setting of the VR experience made patients more comfortable in and less scared of the real medical space around them. Patients also responded positively to the DocVR content, reporting that they enjoyed the experience and found the game interesting. The game was very immersive, with a vast majority of patients reporting that they felt like they were “really there.” The hardware itself was deemed both comfortable and user-friendly.

The authors found only 3 other studies that used VR as an exposure tool to alleviate pediatric preoperative anxiety. However, the VR experience for all 3 of these studies was an immersive guided tour of the operating theater [25,26,34]. While 1 of these 3 VR tours was conducted by another familiar childhood cartoon [26], the interactive nature of the *Doc*VR game allows patients to take an active role in medical play and provides an important addition to VR’s cognitive load that ultimately is the key to alleviating cognitive states like pain and anxiety [35,36].

Patients’ reports confirmed the excellent quality of the game. Many of the patients agreed that the virtual environment looked “real,” and a large majority reported feeling like they were really in the virtual world. Patients were both interested and engaged, and a majority of patients said the game was also interesting compared to other video games they had played.

### Effectiveness of DocVR

Patients experienced a strong statistically significant decrease in anxiety following *Doc*VR gameplay*.* After playing the *Doc*VR experience, patients also reported more positive overall affect, less fear, and less time spent thinking about their upcoming procedure. The VR experience also made patients more comfortable in the hospital, with 1 patient, when asked his favorite part of the game, saying, “I liked the patient going to sleep, so [the patient] didn’t feel any pain.” This response demonstrates the power of familiarizing pediatric patients with their upcoming procedures, putting them at ease for when the time for the procedure comes. *Doc*VR also made patients feel “Good” and “Happy.”

While the hardware required for VR, including headsets and controllers, is rapidly deemed out of date due to the rapid expansion of VR innovation and development, the software nature of *Doc*VR makes it an effective product that can stay relevant as the VR world surges forward.

### VR Age Restrictions

There is still some debate around the appropriate age for the use of VR in pediatrics. Most VR headset manufacturers (Sony, Google, Samsung, HTC, Oculus) recommend that their products should not be used by individuals younger than 12-14 years of age. The Google Daydream, the headset used in this study, “should not be used by children under the age of 13,” according to the manufacturer’s website [37]. The concerns for pediatric use can generally be thought of as being related to (1) legal and liability concerns and (2) safety concerns. The 1998 Children’s Online Privacy Protection Act (COPPA) governs the collection and use of data generated by children under the age of 13 years by websites, mobile applications, and smart devices [38]. COPPA created rules for privacy policies, data collection, parental consent, and parent access to and control over a child’s data. Importantly, COPPA does not prohibit the collection of data but, rather, creates a regulatory framework for how the data should be collected [39]. Since many VR headsets include creating user accounts, the COPPA rules apply to them. In our study, we did not have study patients create user accounts. From a regulatory standpoint, although a handful of VR applications have been approved by the US Food and Drug Administration, the regulatory environment is still evolving. In 2017, the Food and Drug Administration released its Digital Health Innovation Action Plan [40] and, in 2020, held a public workshop to convene industry, investigators, providers, and regulators to discuss best evaluation practices for medical extended reality [41]. When VR is used for distraction or entertainment, such as in this study, it is not considered a medical device [42].

Safety concerns for pediatric VR have focused on the physical fit of the device (headsets may be too big for some young children and, if not worn correctly, can cause physical discomfort), safety around device use (such as accidentally bumping into objects in the real world), and health impacts of VR. Some users experience dizziness, headaches, and motion sickness during or after VR, but over 15 years of pediatric studies have demonstrated the overall safety and minimal side effects of VR in children as young as 6 years [21,22,43-46]. In our study, which included 42 patients under the age of 12 years, no patients reported any symptoms after using the VR headset. While there have been concerns about the possible impact of VR on vision, the American Academy of Ophthalmology states that, although there have been no long-term studies, there is little reason to be worried about VR’s impact on eye development or function [47]. A 2019 study of 50 children aged 4-10 years showed no significant impact on visuomotor function after VR use [48]. Finally, from a parenting and development standpoint, the American Academy of Pediatrics has stated that the 2016 media use guidelines [49] apply to a variety of media, including VR [50]. Parents should make sure content is developmentally appropriate for their children; that they adhere to overall screen time recommendations; to balance media use with media-free time for physical activity, education, play, sleep, and bonding; and whenever possible, to coview or coexperience media together. Various methods of delivering VR have been implemented to validate these data and to ensure that VR is a valid, ethical, and safe option for all pediatric patients [51].

### Limitations

One limitation of this study is the small sample size that, though not uncommon in feasibility research, may limit the generalizability of the conclusions.

This pilot did not utilize a control group; all patients were given the option to play VR. Therefore, researchers were unable to compare the effects of playing *Doc*VR with the anxiety of children who did not play the game. Additionally, the observed positive impact of the *Doc*VR experience on the patients could have been confounded by the positive impact of research assistants’ general active engagement with patients.

Another limitation to the pilot is that not all patients completed the entire *Doc*VR experience. There were 2 scenarios that would have prematurely terminated the VR experience: (1) the child deciding to take the headset off or (2) the child being called to the preoperative area by a health care provider.

A premature end to the VR experience resulted in incomplete playing time and therefore, an incomplete assessment of *Doc*VR for managing pain and anxiety (dose effects). The average time spent playing *Doc*VR may have been higher if the pilot had been conducted in a more controlled environment. However, because the pilot was conducted in a dynamic setting, parts of the protocol were adapted to accommodate clinical care. 

In the scenario where children were quickly called back to the preoperative area by the health care provider, there was insufficient time for the post-VR survey to be administered. The results reported in this paper are thus impacted by incomplete data collection.

Other potential confounds include that 90% (46/51) of patients were familiar with Doc McStuffins*,* and 29% (15/51) of patients had played VR before. This prior exposure may have primed patients to benefit from a pre-existing relationship or experience with Doc, enjoying the *Doc*VR experience, or other content and technology confounds. Nonetheless, this patient population could be representative of a young, TV-watching population, indicating that familiarity may, in fact, be a beneficial component of relieving pediatric preoperative anxiety.

### Conclusions

This is the first feasibility study on the use of *Doc*VR to ease preoperative anxiety in pediatric patients and one of a few studies ever to use VR to address preoperative anxiety in pediatric patients prior to surgery. These results demonstrate the potential utility of VR and, particularly, *Doc*VR in the preoperative space. The present study capitalized on the fusion of familiar and lovable characters with immersive VR gameplay to transform the patient preoperative experience from unpleasant and potentially frightening to fun. Given that health care institutions continue to use patient and family satisfaction as a metric of success [52], digital therapeutic solutions that patients find enjoyable and distracting prove to be worthwhile investments. Furthermore, developers in pediatric health care would benefit from collaborating with children’s media companies in order to capitalize upon pre-established characters and the emotional experience or relationship between children and best-loved characters. Continued research on emerging technologies and VR experiences is essential to ensure that science and an evidence base drive clinical interventions for pain and stress management in a pediatric environment.

## Abbreviations

ASC: ambulatory surgery center
CCLS: Certified Child Life Specialist
CHLA: Children’s Hospital Los Angeles
COPPA: 1998 Children’s Online Privacy Protection Act
DocVR: “Doc McStuffins: Doctor for a Day” Virtual Reality Experience
FAS: Facial Affective Scale
OR: operating room
VAS: visual analogue scale
VR: virtual reality
